# Supplementary material for: The causal role of circulating inflammatory markers in osteoporosis: a bidirectional Mendelian randomized study
Source: Front Immunol. 2024 Jul 18;15:1412298. doi: 10.3389/fimmu.2024.1412298 (PMC11291241; doi:10.3389/fimmu.2024.1412298)
Supplement: Supplementary file 1 [file DataSheet_1.zip › supplementary material/OSTEOPOROSIS-CIM/GCST90274768.tsv.gz_scatter.pdf]

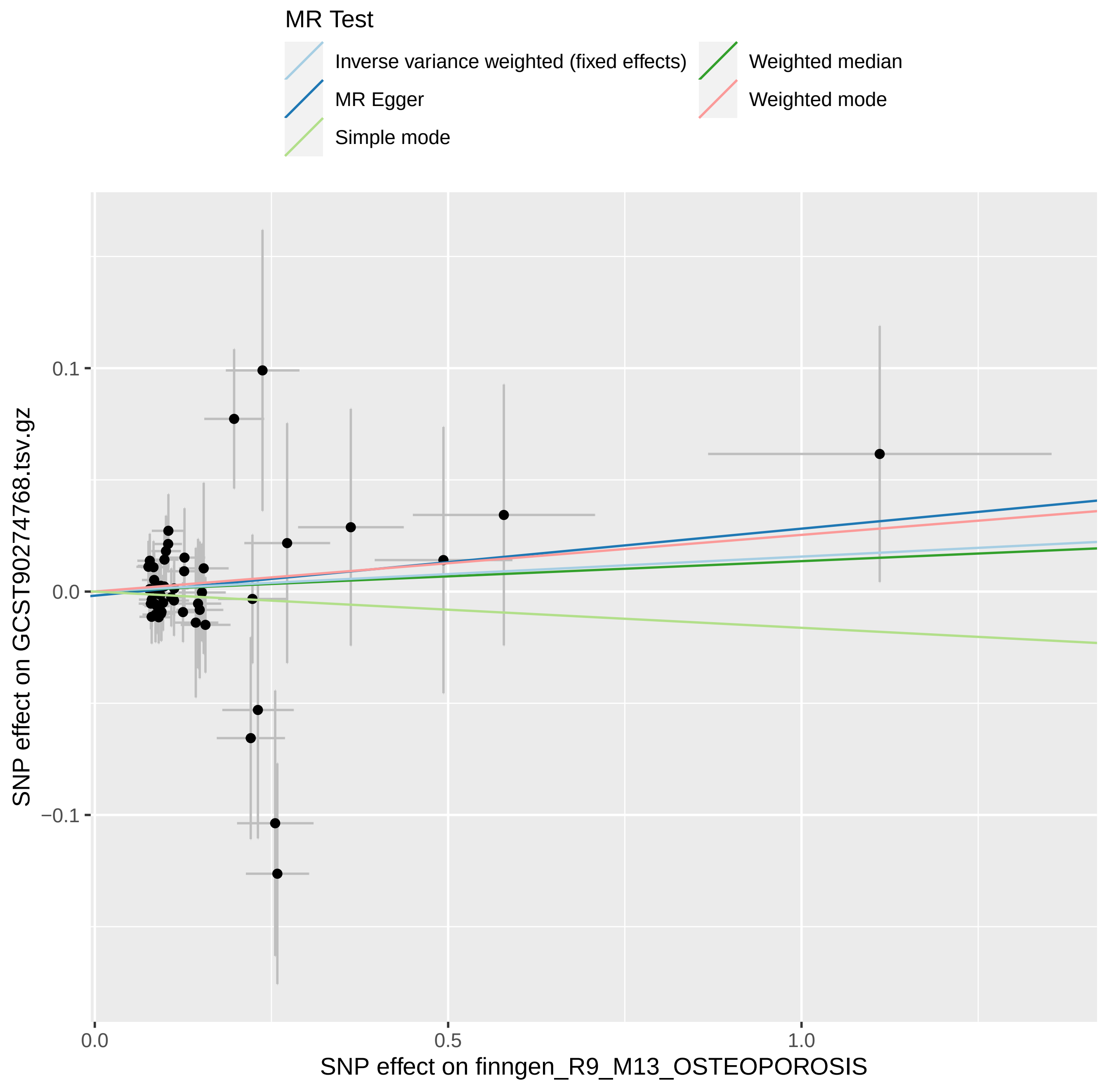
MR Test
Inverse variance weighted (fixed effects)
MR Egger
Simple mode
Weighted median
Weighted mode
0.1
0.0
−0.1
0.0
0.5
1.0
SNP effect on GCST90274768.tsv.gz
SNP effect on finngen_R9_M13_OSTEOPOROSIS
